# Evidence for Cognitive Aging in Midlife Women: Study of Women’s Health Across the Nation

**DOI:** 10.1371/journal.pone.0169008

**Published:** 2017-01-03

**Authors:** Arun S. Karlamangla, Margie E. Lachman, WeiJuan Han, MeiHua Huang, Gail A. Greendale

**Affiliations:** 1 Divison of Geriatrics, David Geffen School of Medicine at UCLA, Los Angeles, California, United States of America; 2 Department of Psychology, Brandeis University, Waltham, Massachusetts, United States of America; Texas Technical University Health Sciences Center, UNITED STATES

## Abstract

Although cross-sectional studies suggest that cognitive aging starts in midlife, few longitudinal studies have documented within-individual declines in cognitive performance before the seventh decade. Learning from repeat testing, or practice effects, can mask the decline in younger cohorts. In women, the menopause transition also affects test performance and can confound estimates of underlying decline. We designed this study to determine if, after controlling for practice effects, the menopause transition, and the symptoms associated with it, there is evidence of cognitive aging in midlife women. We used data from a longitudinal observational study in 2,124 participants from the Study of Women’s Health Across the Nation. Outcomes examined were scores on annual tests of processing speed, verbal episodic memory (immediate and delayed), and working memory. To reduce the impact of practice effects and of the menopause transition, we used the third cognition testing visit as the baseline. Average age at this baseline was 54 years, and the majority of the women were postmenopausal; half the cohort was 2 or more years beyond the final menstrual period. There were 7,185 cognition assessments with median follow-up time of 6.5 years. In mixed effects regression, adjusted for practice effects, retention, menopause symtoms (depressive, anxiety, vasomotor, and sleep disturbance), and covariates, scores on 2 of 4 cognition tests declined. Mean decline in cognitive speed was 0.28 per year (95% confidence interval [CI] 0.20 to 0.36) or 4.9% in 10 years, and mean decline in verbal episodic memory (delayed testing) was 0.02 per year (95% CI: 0.00 to 0.03) or 2% in 10 years. Our results provide strong, longitudinal evidence of cognitive aging in midlife women, with substantial within-woman declines in processing speed and memory. Further research is needed to identify factors that influence decline rates and to develop interventions that slow cognitive aging.

## Introduction

Although decline in cognitive functioning is common in older ages [1,2], there is controversy about whether there is significant decline in cognitive abilities in midlife. Inverse associations between age and cognitive functioning have been seen in cross-sectional analyses of data from middle-aged adults [3,4]; yet within-person longitudinal declines in cognitive performance have not been consistently documented in those under 60 years of age [5,6]. One large study that assessed cognitive performance 3 times over 10 years, did demonstrate longitudinal declines in cognitive performance in midlife, albeit at a slower rate than that of older adults [7].

Yet, in midlife women going through the menopause transition (MT), at least 2 cohorts found no evidence of cognitive aging; instead they documented significant *improvements* in performance over multiple years [8,9]. Similar *improvements* in cognitive performance in midlife have been documented in the Baltimore Longitudinal Study of Aging, and attributed to learning or practice effects from repeat testing [10]. The phenomenon of learning from repeat testing has long been recognized as hampering the estimation of underlying longitudinal change in cognitive performance, and is thought to lead to underestimation—even masking—of true decline [4,6,10,11,12].

Practice effects are largest at first re-testing and diminish significantly with further re-testing [13,14]. We therefore, undertook an analysis of longitudinal cognitive performance data in midlife women from the Study of Women’s Health Across the Nation (SWAN), after excluding data from SWAN’s first 2 cognition testing visits to reduce the impact of practice effects. It has also been suggested that the MT and associated symptoms may impair performance [8,14,15]; both may have confounded previous studies that failed to find evidence of cognitive aging in midlife women. The majority of the SWAN cohort was post menopausal at 3^rd^ cognition testing; thus commencing analysis at 3^rd^ testing reduces effects of the MT on estimates of cognitive performance trajectories. We hypothesized that, after largely eliminating practice effects by initiating our analysis at the 3^rd^ third testing occasion, and explicitly controlling for the MT and associated symptoms, midlife women would indeed show gradual declines in cognitive performance.

## Methods

SWAN is a community-based, longitudinal study of midlife women. Entry requirements were: 42 to 52 years of age; intact uterus; at least one ovary; no estrogen use; and at least one menstrual period in the 3 months prior. Seven study sites together recruited 3302 women [16]; the baseline visit occured in 1996/97, and participants were followed annually thereafter. SWAN participants provided written informed consent, and approval was obtained from Institutional Review Boards at each of the seven SWAN clinical sites and the SWAN coordinating center—Massachusetts General Hospital, Boston, MA; Rush University Medical Center, Chicago, IL; University of Michigan, Ann Arbor, MI; University of California, Los Angeles, CA; Albert Einstein Medical College, New York, NY; Kaiser Permanente Northern California, Oakland, CA; University of California, Davis, CA; and University of Pittsburgh, Pittsburgh, PA. Cognition testing was first administered at the 4^th^ follow-up to 2709 women, and repeated in 6^th^ and subsequent visits up to the 12^th^ follow-up, except that only half the cohort was tested in the 8^th^ follow-up and the remainder in the 9^th^, and there was no cognition testing in the 11^th^ follow-up.

### Study Sample

Of the 2709 women in the SWAN cognition cohort, 2168 (80%) had testing at 3 or more visits—an inclusion criterion for this analysis, which used the 3^rd^ cognition testing as baseline. Because only 21 (<1%) were from the Hudson County (New Jersey) site, they were excluded; an additional 23 were excluded because of a stroke before their 3^rd^ test, leaving a sample of 2124. Because cognitive aging may accelerate after the menopause [17], we created a subsample of 1224 women whose date of final menstrual period (FMP) was known; FMP date may be unknowable due to interim hysterectomy and/or use of exogenous sex hormones.

### Measurements

#### Outcomes

Cognitive processing speed was assessed with the the symbol digit modalities test (SDMT), in which participants match numbers to symbols in a specified time period [18]; score range, 0–110. Verbal episodic memory was evaluated using the East Boston Memory test (EBMT) [19]: Respondents recall story elements from a paragraph read to them, immediately and after ~10 minutes delay; score range, 0–12. Working memory, the ability to manipulate information held in memory, was assessed by digit span backwards (DSB) [20]: Participants repeat strings of single-digit numbers backwards, with 2 trials at each string length, increasing from 2 to 7, stopped after errors in both trials at a string length, and scored as the number of correct trials (range, 0–12).

#### Covariates

At SWAN baseline, questionnaires collected age, race/ethnicity, and education. Annually administered questionnaires assessed financial hardship (difficulty paying for basics), diabetes mellitus, sex hormone use, interim hysterectomy and/or bilateral oophorectomy, MT stage (premenopausal: no change in menses regularity, early perimenopausal: menses within the prior 3 months but less predictable, late perimenopausal: > 3 months but < 12 months of amenorrhea, postmenopausal: ≥12 months without menses, and indeterminate because of premenopausal hysterectomy or use of sex steroid hormones before the MT is completed), and FMP date. The Center for Epidemiologic Studies Depression (CES-D) Scale quantified depressive symptoms [21], and coded present if in the top quartile (≥13). Anxiety symptoms were assessed using the SWAN anxiety score [22], and coded present if in the top quartile (≥7). Sleep disturbance was assessed using an abbreviated Pittsburgh Sleep Quality Index, and coded present if either difficulty falling asleep, waking up several times, or waking up earlier than planned with inability to fall asleep again were reported for ≥3 nights per week [23]. Vasomotor symptoms were coded present if any of hot flashes, cold sweats, or night sweats occurred ≥6 days per week [22].

### Statistical Analysis

After excluding scores from the first 2 cognition testing occasions, we examined LOESS-smoothed plots of cognition scores as a function of ‘time from FMP’ (negative for dates before the FMP and positive for dates after; a proxy for ovarian aging) and ‘time elapsed since the 3rd cognition testing’ (a measure of chronological aging). Time from FMP more closely captures biological aging in a midlife woman, because of the large changes around the FMP not only in sex hormone levels but also in multiple other physiological markers [24,25,26]. At least one prior study found that cognitive aging accelerates after the menopause [17].

**Fig 1:**
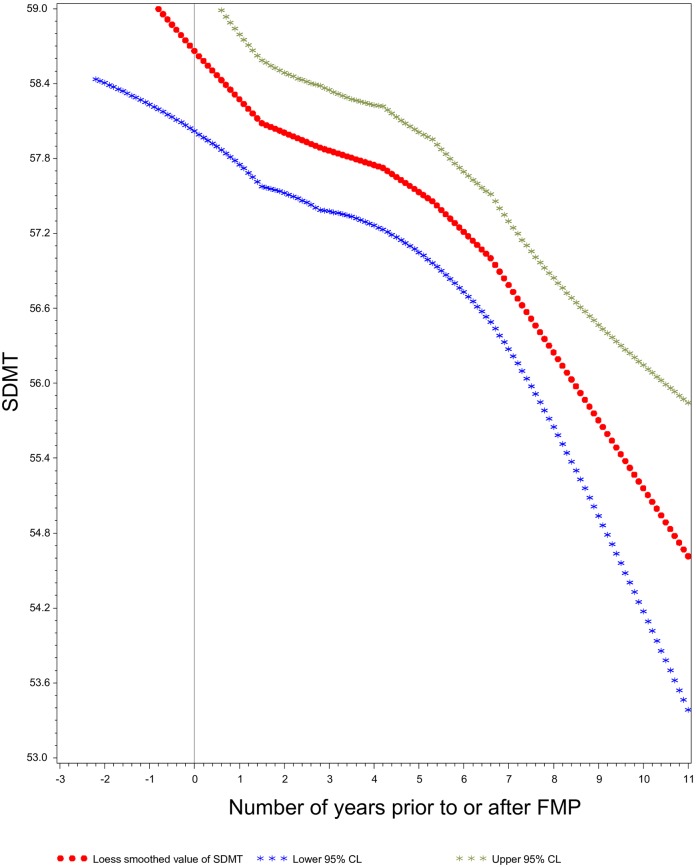
Symbol Digit Modalities Test Scores as Function of Time Prior to and After the Final Menstrual Period. LOESS Smoothed Plot of Scores on Symbol Digit Modalities Test (SDMT), relative to time prior to and after the Final Menstrual Period (FMP), an assessment of the relation between ovarian aging and cogntive processing speed.

**Fig 2:**
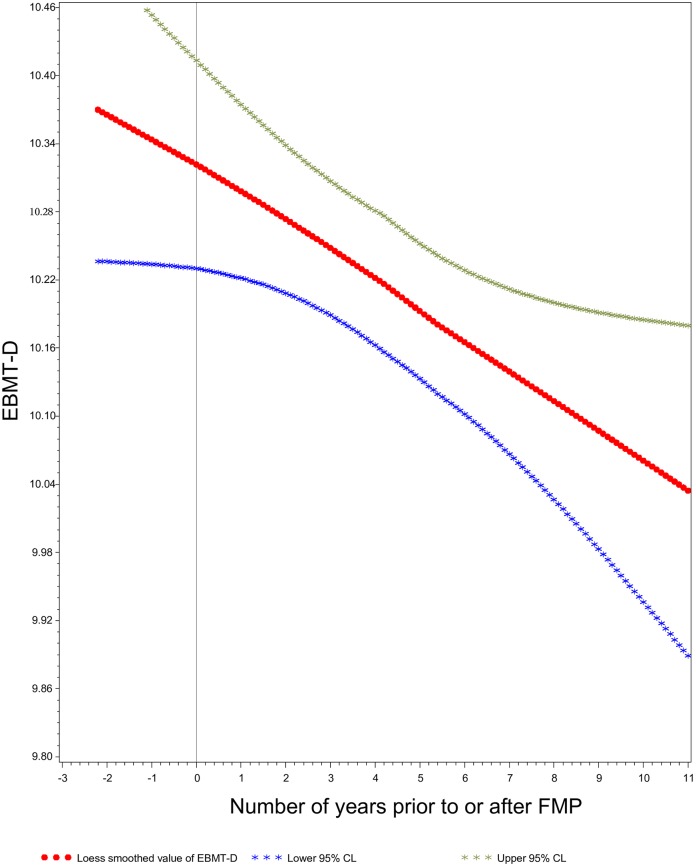
East Boston Memory Test Delayed Recall Scores as a Function of Time Prior to and After the Final Menstrual Period. LOESS Smoothed Plot of Scores on East Boston Memory Test Delayed Recall (EBMT-D) relative to time prior to and after the Final Menstrual Period (FMP), an assessment of the relation between ovarian aging and verbal episodic memory.

**Fig 3:**
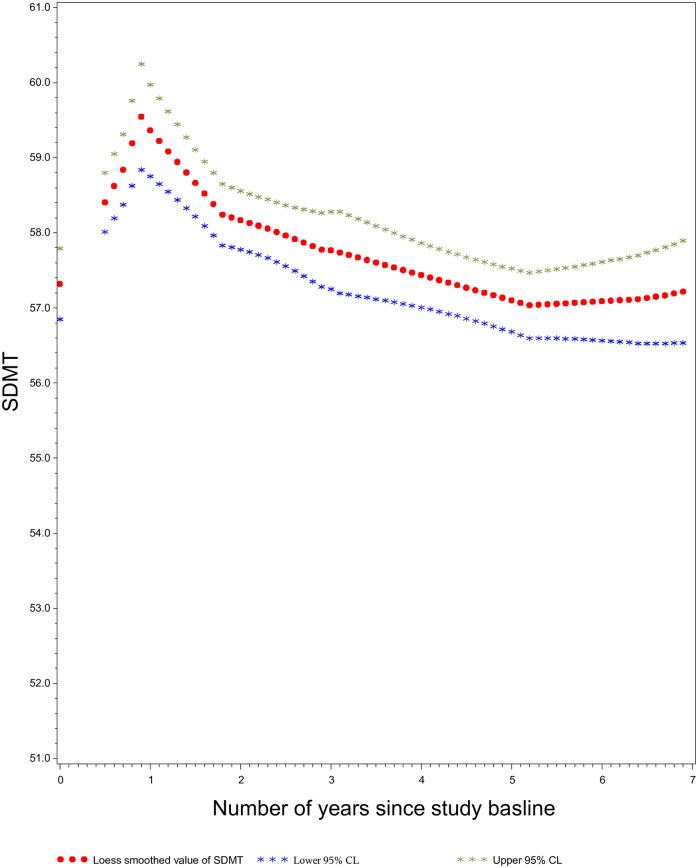
Symbol Digit Modalities Test Scores as Function of Chronological Aging. LOESS Smoothed Plot of Scores on Symbol Digit Modalities Test (SDMT), relative to time elapsed since study baseline (for this analysis, the 3^rd^ cognitive testing occasion), an assessment of the relation between chronological aging and cogntive processing speed.

The LOESS plots showed steady declines in the mean values of each of the 4 cognition scores as time from FMP increased (SDMT: Fig 1, EBMT-Delayed: Fig 2, EBMT-Immediate and DSB: not shown). In contrast, the LOESS plots against ‘time since 3^rd^ testing occasion’ showed gradual decline only in SDMT (Fig 3), and not in the other scores (data not shown), and revealed a persistent learning/practice effect from the 3^rd^ to 4^th^ testing in all scores.

We fit linear growth curves to the repeated measurements of each cognition score as function of time from FMP, allowing for a residual practice/learning effect from the 3^rd^ to 4th testing, and censoring observations after an incident stroke. We used linear mixed effects regression with a random intercept at the participant level to account for clustering of repeated observations from the same woman. Covariates, chosen for known or hypothesized relation to cognitive performance, were modeled as fixed effects on the level (intercept), and included the following time-fixed variables: age at FMP, education (≤high school, some college, baccaleurate, post-graduate), race/ethnicity (Black, Chinese,Japanese White), testing language (English, Cantonese Chinese, Japanese), difficulty paying for basics (no hardship, somewhat hard, very hard, refused), use of sex steroids prior to the 3^rd^ cognition testing (yes/no), and the total number of cognition assessments per participant (proxy for characteristics that affect retention in the study, strongly associated with cognitive performance in older cohorts [1,27,28]). The ‘age at FMP’ covariate controls for differences in cognition performance due to any chronological aging effects prior to the FMP, and isolates biological aging effects related to the MT. Models also included these time-varying covariates: learning/practice effect (0 for the 3rd testing and 1 for ≥4^th^), use of sex steroid hormone therapy (yes/no), bilateral oophorectomy before natural menopause (yes/no), diabetes (yes/no), and MT-associated symtoms (depressive, anxiety, sleep disturbance, and vasomotor). Analyses were conducted using complete cases (no covariates missing).

To examine the relation between cognitive performance and chronlogical aging, we ran parallel models that fit linear growth curves to the cognition scores as a function of time since 3^rd^ cognition testing, with all the same covariates except that age at FMP was replaced by age at the 3^rd^ cognition testing (to capture cross-sectional age differences in cognition). We included one additional time-varying covariate, MT stage at the time of testing, to remove effects of the MT, and isolate chronological aging effects. Because this analysis did not require an FMP date, it was done in the larger sample of 2124 participants who met study entry criteria (except not requiring known date of FMP).

## Results

The study sample was similar to the SWAN cognition cohort (Table 1). At the 3^rd^ cognition testing (baseline for this analysis), median age was 54 years (interquartile range 52 to 56), and the majority of the women were post-menopausal. Nearly half were Caucasian, and the majority were tested in English. About a quarter had post-graduate education. The FMP subsample was very similar to the study sample (Table 1); at the baseline for this analysis, half of these women were at least 2 years past the FMP.

**Table 1:** Descriptive Statistics^1^ for the Study Sample and the FMP Subsample compared to the SWAN Cognition Cohort.

Participant Characteristics (Number of participants)	Study Sample^2^ (2,124)	FMP Subsample^3^ (1,224)	SWAN Cognition Cohort^4^ (2,709)
Age at third cognition testing^5^ (years)	54.0 (3.0)	54.0 (3.0)	-
Age at FMP (years)	-	52.0 (2.6)	-
Race/ethnicity			
White	1045 (49.2%)	551 (45.0%)	1276 (47.1%)
Black	624 (29.4%)	376 (30.7%)	771 (28.5%)
Chinese	214 (10.1%)	146 (11.9%)	235 (08.7%)
Japanese	241 (11.3%)	151 (12.3%)	258 (09.5%)
Hispanic	0	0	169 (06.2%)
SWAN clinical site			
Boston, MA	352 (16.4%)	212 (17.3%)	397 (14.6%)
Chicago, IL	299 (14.1%)	184 (15.0%)	370 (13.7%)
Detroit, MI	382 (18.0%)	226 (18.5%)	447 (16.5%)
Los Angeles, CA	415 (19.5%)	223 (18.1%)	453 (16.7%)
Hudson County, NJ	0	0	248 (09.1%)
Oakland, CA	369 (17.4%)	231 (18.8%)	417 (15.4%)
Pittsburgh, PA	307 (14.5%)	148 (12.1%)	377 (13.9%)
Education level			
High school or less	396 (18.7%)	239 (19.7%)	606 (22.6%)
Some college	681 (32.2%)	397 (32.6%)	869 (32.3%)
Baccalaureate	481 (22.8%)	279 (23.0%)	568 (21.1%)
Postgraduate	555 (26.3%)	301 (24.7%)	644 (24.0%)
Menopause transition (MT) stage at time of 3^rd^ cognition testing			
Premenopause	38 (1.8%)	23 (1.9%)	-
Early perimenopause	461 (21.7%)	309 (25.2%)	-
Late perimenopause	196 (9.2%)	164 (13.4%)	-
Post menopause not taking sex steroids	1057 (49.8%)	710 (58.0%)	-
Postmenopause taking sex steroids	75 (3.5%)	18 (1.5%)	-
Indeterminate MT stage	295 (13.9%)	0 (0.0%)	-
Use of sex hormones prior to third testing	562 (26.5%)	30 (2.5%)	
Financial hardship at third testing			
Not hard paying for basics	1508 (71.0%)	842 (68.8%)	-
Somewhat hard	465 (22.0%)	296 (24.2%)	-
Very hard	109 (5.1%)	64 (5.2%)	-
Refused	42 (1.9%)	22 (1.8%)	-
Diabetic at 3^rd^ cognition testing	193 (9.1%)	116 (9.5%)	
Depressive symptoms 3^rd^ cognition testing	448 (21.1%)	248 (20.3%)	
Anxiety symptoms at 3^rd^ cognition testing	671 (31.6%)	370 (30.2%)	
Sleep disturbance 3^rd^ cognition testing	627 (29.5%)	328 (26.8%)	
Vasomotor symptoms 3^rd^ cognition testing	556 (26.2%)	312 (25.5%)	
Language of cognition testing			
English	1945 (91.6%)	1101 (90.0%)	2372 (87.6%)
Cantonese Chinese	88 (4.1%)	57 (4.6%)	82 (3.0%)
Japanese	91 (4.3%)	66 (5.4%)	102 (3.8%)
Spanish	0	0	153 (5.6%)
Cognition test scores 3^rd^ cognition testing			
Symbol Digit Modalities Test	57.3 (11.1)	57.2 (11.3)	-
East Boston Memory Test—Immediate	10.4 (1.7)	10.3 (1.6)	-
East Boston Memory Test—Delayed	10.2 (1.8)	10.2 (1.8)	-
Digit Span Backwards	6.88 (2.3)	6.86 (2.3)	-
Number of cognition assessments, starting with 3^rd^ testing occasion	3.4 (0.9)	3.4 (0.9)	

^1^ Mean (standard deviation) for continuous variables; number (percentage) for categorical variables

^2^ Women from the SWAN Cognition Cohort who met the requirements for inclusion in the current analysis; the primary reason that women in the SWAN Cognition Cohort were excluded from this study sample was that they had cognition testing on 2 or fewer visits (see Methods).

^3^ Subset of the study sample for whom the date of the final menstrual period (FMP) was known.

^4^ SWAN enrolled 3302 women at baseline. The study initiated cognitive performance testing at the 4^th^ follow-up visit. The SWAN Cognition Cohort consists of women who were still participating in SWAN at or after the 4^th^ follow-up and who agreed to undergo cogntive testing.

^5^ The 3^rd^ administration of SWAN cognitive tests served as the baseline for this analysis.

In the study sample and FMP subsample, the mean number of cognition testing occasions available for analysis was 3.4, but the majority of women participated in all 4 cognitive visits (at the 7^th^, either 8^th^ or 9^th^, 10^th^, and 12^th^ follow-ups). There was a total of 7,185 repeated assessments in the study sample, and 4,163 in the FMP subsample. Some women did not complete all 4 cognition tests at every testing occasion: in the study sample, there were 31 missing SDMT scores, 2 missing EBMT immediate recall, 6 missing EBMT delayed recall, and 171 missing DSB scores; corresponding missing numbers in the FMP subsample were 18, 0, 2, and 94. The median length of follow-up was 6.5 years (interquartile range 5.2 to 6.8) in the study sample, and 6.5 years (interquartile range 5.6 to 6.8) in the FMP subsample.

In linear, mixed effects regression in the FMP subsample, adjusted for age at the FMP, practice (learning) from the 3^rd^ to the 4^th^ testing, and retention, SDMT scores decreased on average by 0.27 per year (p < .0001), and EBMT delayed recall scores decreased on average by 0.02 per year (p = 0.01). These declines persisted after additional adjustment for education, race/ethnicity, testing language, clinical site, financial hardship, oophorectomy, sex hormone use, diabetes, and symptoms of depression, anxiety, sleep disturbance, and vasomotor instabily, although the decline in EBMT-delayed recall scores became marginally significant (Table 2, ovarian aging model). Between-women difference in SDMT score by age at FMP (0.31 decline per year) was nearly identical in magnitude to the longitudinal aging effect (0.28 decline per year). There were no cross-sectional or longitudinal aging effects seen for the other 2 cognition test scores: EBMT immediate recall and DSB (Table 2).

**Table 2:** Adjusted, Annualized Rates of Change^1^ in Cognition Test Scores: Results of Linear Mixed Effects Regressions.

	Symbol Digit Modalities Test	East Boston Memory Test—Immediate Recall	East Boston Memory Test—Delayed Recall	Digit Span Backwards
**Ovarian Aging Model**^2^				
** Age at final menstrual period (FMP)** (Between-women differences)	**-0.31** (-0.50, -0.11)	0.01 (-0.02, +0.04)	0.01 (-0.02, +0.04)	-0.01 (-0.05, +0.03)
** Time since final menstrual period** (Within-woman, longitudinal)	**-0.28** (-0.36, -0.20)	-0.01 (-0.02, +0.01)	**-0.02** (-0.03, 0.00)	0.00 (-0.02, +0.02)
**Chronological Aging Model**^3^				
** Age at 3**^**rd**^ **testing** (Between-women, cross-sectional)	**-0.54** (-0.67, -0.40)	0.00 (-0.02, +0.02)	-0.01 (-0.03, +0.01)	**-0.03** (-0.06, 0.00)
** Time since 3**^**rd**^ **testing** (Within-woman, longitudinal)	**-0.25** (-0.32, -0.18)	0.00 (-0.02, +0.02)	0.00 (-0.02, +0.01)	0.00 (-0.02, +0.02)

^1^ Annualized slope (95% confidence interval)

^2^ The ovarian aging model quantifies the change in cognitive test performance in relation to time from FMP (time prior to the FMP takes on a negative value and time after the FMP a positive value—see Methods for details). Model adjusted for age at FMP, practice/learning effects, retention, race/ethnicity, education level, language of testing, financial hardship, use of sex hormones, bilateral oophorectomy, diabetes, depression, anxiety, vasomotor symptoms, sleep disturbance, and study site, using 4,162 observations from 1,223 women (after dropping one observation because of missing covariates).

^3^ The chronological aging model quantifies the change in cognitive test performance in relation to time elapsed since the 3rd cognition testing (baseline for the current analysis). Model adjusted for age at 3^rd^ testing, practice/learning effects, retention, race/ethnicity, education level, language of testing, financial hardship, use of sex hormones, bilateral oophorectomy, diabetes, depression, anxiety, vasomotor symptoms, sleep disturbance, study site, and menopause transition stage at time of testing, using 7,189 observations from 2,121 women (after dropping 6 observations because of missing covariates).

There were also strong practice effects in SDMT, but not in the other 3 test scores: mean SDMT learning from 3^rd^ to 4^th^ testing was 0.62 (p = 0.01). In addition, there were retention effects seen in 3 of the 4 tests; scores were higher in those who were tested more often: SDMT (p = 0.0001), EBMT delayed recall (p = 0.04) and DSB (p = 0.06).

The second set of models examined test scores as a function of chronological aging—calendar time since the 3^rd^ cognition testing—and yielded similar findings. Adjusted only for age at the 3^rd^ testing, practice from the 3^rd^ to 4^th^ fourth testing, and retention, SDMT scores decreased on average by 0.24 per year (p < .0001). This decline persisted in the fully adjusted model (Table 2, chronological aging model). Between-women difference in SDMT score by age at 3^rd^ testing was more than double the longitudinal aging effect: 0.54 vs. 0.25 decline per year (Table 2). There were no longitudinal aging effects seen for the other 3 cognition tests, but there was a cross-sectional age effect on DSB scores (Table 2). As before, there were strong practice (0.60; p = 0.001) and retention effects (p<0.0001) on SDMT scores. There was also a positive retention effect on EBMT immediate recall (p = 0.02) and EBMT delayed recall (p = 0.0005).

To remove residual confounding by persisting practice effects, we conducted a sensitivity analysis in which we ran the mixed effects models after dropping data from the 3^rd^ cognition testing and allowing for a practice effect from 4^th^ to 5^th^ testing. Longitudinal aging estimates from the fully adjusted models were somewhat larger: SDMT declined 0.35 per year (95% confidence interval [CI]: 0.24, 0.46) in the ovarian aging model (2,926 observations from 1,130 women) and 0.25 per year (95% CI: 0.15, 0.36) in the chronological aging model (5,041 observations from 1,963 women).

## Discussion

As hypothesized, after controlling for practice effects, the MT and MT symptoms, midlife women did show longitudinal declines in cognitive performance, mainly in processing speed. The average, within-woman rate of decline (longitudinal aging effect) in processing speed was essentially identical to the average, between-women difference by age at time of FMP (0.28 per year vs. 0.31 per year). However, as in previous studies [7], cross-sectional differences by chronological age at time of testing were substantially larger than longitudinal aging effects (0.54 per year vs. 0.25 per year), likely because between-women differences in ovarian age were not completely eliminated by controls for MT stage.

Previous studies have found that cognitive procesing speed is especially sensitive to early changes [29,30,31]. We found longitudinal declines in both processing speed and verbal memory (delayed recall) in this study. The estimated decline rates translate to a 10-year reduction of approximately 0.25 standard deviations (SD) of the baseline score or 4.9% of the mean baseline score in processing speed, and 0.11 SD of the baseline score or 2.0% of the mean baseline score in delayed recall. These rates are similar to the 10-year longitudinal decline of 3.6% in reasoning score seen in 45–49 year old women in the Whitehall cohort [7]. Consistent with previous work, we also did not see longitudinal declines in immediate recall and working memory; however, more sensitive measures of episodic and working memory might indeed show declines in midlife.

Although there is some evidence that circulating estrogen might protect premenopausal women from cognitive aging [32,33], we did not see a sharp acceleration of cognitive decline during or after the menopause transition (Figs 1 and 2). Instead, the rates of longitudinal decline in SDMT scores were nearly identical regardless of whether time was indexed to the date of the FMP (ovarian aging model) or measured from study baseline (chronological aging model). However, MT-related declines in circulating estradiol level start 2 years *before* the FMP, and declines in other estrogen-dependent biological systems, such as bone, commence well before the FMP [26,34]. Because 75% of participants in the current analysis were 52 years of age or older at study baseline and the mean age at FMP was 52, the vast majority were “past” the time when an estradiol-related inflection in the cognitive performance trajectory might occur. The likelihood of such an inflection in cognitive performance trajectory prior to the FMP is supported by our finding that between-women differences by age at FMP were smaller than between-women differences by age at time of testing.

As in older cohorts, we also saw a retention effect in this midlife cohort: Cognitive performance was better in women who stayed in the study longer, although attrition was not primarily due to death in this cohort. At least one other study found similar differences by retention in cognitive performance in the 6^th^ decade, but concluded that selective retention did not bias estimates of longitudinal cognitive decline [35].

Limitations of our study include the inability described above to detect initiation or acceleration of cognitive decline at the time that estrogen starts declining, absence of men from the study, and limited generalizability to women not represented in this study, including those who use sex hormones during the MT, who had a hysterectomy without bilateral oophorectomy prior to natural menopause, who were too ill to participate, from less developed economies, and women from race/ethnicity groups not represented in SWAN.

In conclusion, this study provides good new evidence of cognitive aging in women in midlife, with significant longitudinal declines in both processing speed and verbal memory. Unlike previous longitudinal studies in midlife that were based on 3 or fewer cognition assessments, and could not adequately account for practice effects, we analyzed up to 6 annual or biennial assessements, allowing us to minimize the impact of practice effects and unmask declines. Practice effects are larger in younger, cognitively intact individuals than in older adults [36] and can dominate over the smaller declines in cognitive performance in midlife; more complete elimination of practice effects may show that midlife declines are even steeper.

A decline in processing speed in midlife is not a harbinger of declines in other domains of functioning [37], there are individual differences in cognitive aging, and resilience and compensatory mechanisms can ameliorate the impact of cognitive aging on functioning and well-being [38]. Cognitive aging may also be malleable [39,40]. Further research is needed to determine factors that influence differential rates of decline in cognitive performance with an eye towards developing interventions aimed at slowing cognitive aging.
